# Religion as a domain of exposure assessment in epidemiologic studies: History, meaning, and implications

**DOI:** 10.1016/j.gloepi.2025.100218

**Published:** 2025-09-26

**Authors:** Jeff Levin

**Affiliations:** Institute for Studies of Religion, Institute for Global Human Flourishing, and Medical Humanities Program, Baylor University, Waco, TX 76798, USA

**Keywords:** Assessment, Epidemiology, Exposures, Methodology, Public health policy, Religion

## Abstract

For over a century, research findings have accumulated linking measures of religious identity and practice to rates of morbidity and mortality and other population-health outcomes. These studies have been conducted on every continent, have drawn on population samples of nearly all major religions and denominations, and have investigated associations with numerous overall and cause-specific rates of most major chronic diseases and acute conditions, both physical and psychiatric. Yet despite thousands of published studies and many comprehensive reviews, relatively less attention has been paid to conceptual, theoretical, and policy-related issues, notably what these studies are actually assessing, what resultant findings mean and do not mean, and why they should matter. This has contributed, in part, to the continued contentiousness of this subject within some segments of medicine and epidemiology. In an effort to clarify issues, this commentary outlines (a) the current status of exposure assessment related to religion; (b) the ways that the meaning of empirical findings are occasionally misjudged; (c) possible mediators of putative religious influences on morbidity and mortality; and (d) policy implications of existing findings. The latter includes implications for epidemiologic and population-health research, for clinical medicine, for congregational health promotion and disease prevention programs, for global health development and public health policy, and for human flourishing. In sum, this area of research can make a worthwhile contribution, but the burden is on investigators to ensure that their religious measures are validated and that findings are carefully unpacked in terms of their real-world implications for population health.

## Introduction

For over 130 years, epidemiologic research has linked measures of religiousness, broadly defined, to population-health outcomes, both medical and psychiatric and for both morbidity and mortality [1,2]. This work accumulated in relative obscurity until gaining wider attention in the 1980s [2,3]. Since then, these studies have often elicited controversy and a somewhat skeptical response, some of which derives from skepticism about social and psychosocial epidemiology in general [4], some from concerns about the intrusion of the topic of religion into a field of scientific investigation [5], and some from questions about the methodological sophistication of early studies [6]. Much of that resistance has waned, especially regarding the quality of research methods in the best studies [7], but some still remains. While the value of this research is by now more accepted within clinical medicine [8], there also remains an ongoing lacuna of awareness of the provenance, or origins, of this work and of the larger backstory of population-based studies of religion in relation to health- or disease-related outcomes.

This paper focuses on three tasks, summarizing the scope of what is currently known, what it means, and why it should matter. First, it offers a brief narrative history of efforts to assess religion and religiousness in epidemiologic and other population-based studies of physical or mental health. Numerous comprehensive reviews of these studies have been published over the years, including traditional literature reviews [2,3], systematic reviews [9], and meta-analyses [10] all dating back to the 1980s, and some disease-specific reviews of very recent vintage [11], so the aim is not to repeat this work, but rather to provide a summary specifically of assessment-related issues. Second, this paper discusses what the findings from these studies imply and do not imply about a putative protective or risk effect of measures of religious identity or practice—something that has not been as explicitly focused on by previous reviews. Third, it is suggested that, regardless of epidemiologists' own perspectives regarding the value of religion or faith for themselves or for the general population, there may be significant implications of these research findings that merit attention, including for epidemiologic and populaiton-health resarch, for clinical medicine, for community health promotion and disease prevention programs, for global health development and public health policy, and for human flourishing. This summary, it is hoped, provides a concise introduction to the “epidemiology of religion” [12] and makes the case that this area of research, as outside of the mainstream as it may appear, has scientific plausibility and can contribute to furthering the health of populations.

## History

### Research literature

The earliest published research, mostly from descriptive epidemiology, dates to the 1800s. In 1891, for example, John Shaw Billings observed that Jewish identity or religious affiliation appeared to be a source of fertility, morbidity, and mortality differences between Jews and gentiles [13]. Throughout the first several decades of the 20th Century, statistically significant religious differences were reported from research in perinatal [14], occupational [15], psychiatric [16], and geriatric [17] epidemiology, in terms of morbidity or mortality due to mostly chronic as opposed to acute diagnoses. Studies typically compared incidence, prevalence, or mortality rates across religious or denominational categories [18] or compared those reporting a religious affiliation with those who reported none [19] or compared a religious category (e.g., clergy) with “all others” [20]. Other studies looked at associations between exposures assessing levels of religious participation and rates of physical or psychiatric morbidity [2,3]. These included population-health studies inquiring about construct categories such as public religious behavior (e.g., frequency of religious service attendance [12]), private religious practices (e.g., daily prayer [21]), religious beliefs (e.g., in God [22]) or attitudes (e.g., self-rated religiousness [23]), and religious (e.g., being born again [24]) or mystical (e.g., during meditation [25]) experiences. Among these, decades ago, were nearly a dozen studies conducted by preeminent Johns Hopkins epidemiologist George Comstock, most famously an analysis pointing to regular church attendance as an apparently protective factor against overall and cause-specific mortality using data from an epidemiologic census in Washington County, Maryland [26].

By now, according to the three editions of Oxford University Press' definitive *Handbook of Religion and Health* [1,27,28], results from over 10,000 empirical studies have been published, with upwards of 60 % or more reporting salutary (positive) results, depending upon the disease outcome and population, such that greater religiousness—broadly defined—appears to be associated with lower rates of disease or death [1,29]. As determined by a systematic review of the references in the first two editions of the *Handbook*, this observation holds for heart disease morbidity and mortality, hypertension and cerebrovascular disease, cancer morbidity and mortality, all-causes mortality, self-rated health, pain and somatic symptoms, physical disability, depression and anxiety [29]. As with all epidemiologic findings, this overall observation holds on average and across respective populations, as well as *caeteris paribus* (all things being equal). There are of course exceptions to this general finding, which may be difficult to capture when presenting only a single summary finding. Further, results which do not fit this general pattern may represent inverse findings (i.e., religion as a risk factor) or null or non-associations, and, moreover, existing population findings of an apparently protective effect may themselves be biased and overestimated due to statistical power considerations resulting from the very large population samples utilized in many of these epidemiologic studies which in turn may inflate the chance of observing statistically significant findings [30].

A notable feature of this research is that studies, and positive findings, have emerged from population and community samples located worldwide. Findings suggestive of a salutary impact of religiousness, including spiritually-contexted mind-body practices, on physical or mental health have drawn on samples from numerous countries in every continent (even Antarctica [31]). This includes published research as wide-ranging as studies of patterns of cause-specific mortality among Old Order Amish in three U.S. states [32]; psychiatric morbidity among Evangelicals, Spiritists, and Roman Catholics in Brazil [33]; mortality differentials among Muslims and Orthodox Christians of various ethnicities in Bulgaria [34]; breast cancer incidence among Hindus, Muslims, Christians, Parsis, Jains, and Buddhists in India [35]; hypertension and blood pressure readings among rural and urban Zulus in South Africa [36]; and self-ratings of health status among religious elderly in Japan [37].

As well, as long as 30-plus years ago, according to a much-cited review, diversity in this general finding had been observed across sociodemographic categories (by age, sex, race/ethnicity, social class, marital status, urbanicity, nationality), morbidity and/or mortality outcomes (e.g., due to coronary heart disease, cancer, other chronic and acute conditions, psychiatric diagnoses, psychophysiological markers), and research designs (i.e., cohort, case-control, prevalence, bidirectional, multi-wave panels, quasi-experiments) [38]. Especially large literatures had already emerged by then for religion and cancer [39], heart disease [40], hypertension [41], and psychiatric morbidity [9]. Significantly, this diversity in study samples and positive findings (such that greater religiousness was associated with better physical or mental health or less morbidity or mortality) was also observed across categories of religious identity, including most major faith traditions (e.g., Christian, Jewish, Muslim, Buddhist, Hindu, Zoroastrian, Sikh, Jain, Shinto, Taoist) and Christian denominations (e.g., Roman Catholic, Eastern Orthodox, nearly all Protestant denominations and established sects) [[1], [2], [3],^39^]. Likewise, findings reporting on associations with numerous indicators of religious practice had already appeared throughout this literature, including apparent health impacts of behavioral measures of myriad public and private forms of religious or spiritual engagement. Most typically these were frequency of religious attendance, prayer, Bible study, and other “organizational” (i.e., formal, institutional) and “non-organizational” (i.e., informal, non-institutional) forms of religious involvement [42].

Finally, despite an understandable perception at one time among some researchers that this may be a marginal or even disreputable topic for medical and epidemiologic research—one review referred to religion as “the anti-tenure factor” for academic clinical epidemiologists [43]—studies and reviews featuring religious exposures have been published in leading journals in epidemiology for decades [44]. Among these are *American Journal of Epidemiology*, *Annals of Epidemiology*, *Journal of Clinical Epidemiology*, *International Journal of Epidemiology*, *Journal of Epidemiology and Community Health*, *Social Psychiatry and Psychiatric Epidemiology*, *European Journal of Epidemiology*, and more, as well as many articles in *American Journal of Public Health*.

In the 1980s and 1990s, comprehensive review articles began to appear which identified the parameters of this literature. Various measures of religious identity or practice were found to have exhibited statistically significant associations with psychiatric outcomes, including preventive effects on addictive behavior, suicide, and depression and more ambiguous or complex effects with respect to psychosis and certain other psychopathologies [45]. Protective effects were also observed for morbidity and/or mortality due to almost every major category of chronic disease or physical health diagnosis [2,3]. Besides those noted above (i.e., heart disease, cancer), these included religious differences in or associations with pulmonary, viral respiratory, gastrointestinal, genitourinary, cerebrovascular, and neurological diseases, as well as asthma, diabetes, infections, chronic pain and somatic symptoms, physical disability, and myriad other disease entities and conditions [1]. Significant findings (although not all positive) emerged from major community-based population studies of morbidity or mortality, including the Alameda County (California) [46], Evans County (Georgia) [47], and Tecumseh (Michigan) [48] studies; from longitudinal studies [49], which continue to appear [50,51]; from studies of disability [52]; and from research funded by branches of the U.S. National Institutes of Health [53]. Many of these studies have been from programmatic research, even early on [54]—that is, not from one-off analyses of available datasets, often using small nonprobability samples, typical of earlier research [6]—and much of this work involved the study of racial or ethnic minority populations [55].

### Exposure assessment

Notwithstanding the observation of a generally salutary association, an ongoing issue has been how to address concerns regarding what epidemiologists term exposure assessment [56]. Individual indicators are typically used to assess aspects or dimensions of religious identity, expression, or practice, often without reference to the meaning and context of such measures and whether they truly represent or capture what investigators think they are capturing [57]. Results may then be attributed to an undefined “religiosity” without recognition of the multidimensionality of religious identity and participation. This has been noted by sociologists [58] and psychologists [59] who study religion and by religious and theological scholars [[60], [61], [62]], as well as specifically in epidemiologic context [63,64], but, on the whole, population-health research studies have been limited in how they have been able to address this issue. For decades, epidemiologic studies have relied on such single-item indicators alone or in combination with other religious indicators to create, for example, a summary “index of religiousness” [65] or something similar of a presumably religious nature, even where the individual items tap into substantially different domains or aspects of religious engagement. What specific construct(s) such composite measures are actually assessing is unclear. This is not necessarily by intention nor is it implicitly problematic, but rather may exemplify the constraints which create difficulties in enabling deeper dives into respective religious constructs. A couple of single items may be all that survey space and time considerations allow, especially for a meta-construct (religion) perhaps perceived as marginal compared to other more established biological, environmental, and psychosocial exposures and thus not prioritized in the development of data collection instruments.

A case in point is use of a behavioral measure of the frequency of attending public religious services, the mostly widely used exposure variable in this literature. When using such an indicator, it may be helpful to consider just what it is that may matter here for physical or mental health: is it the absolute level of “exposure” to such services (e.g., at least once per week), or is it one's position above or below what is considered normative or optimal attendance for one's respective faith tradition? This is an issue that would be familiar to those investigating the epidemiologic impact of psychosocial exposures in general—that is, the importance of contextual issues—but, to now, this has not been adequately engaged in epidemiologic studies of religion [66]. Again, this may be understandable given the contingencies of mounting large population-based studies; one can only do so much given structural limitations. Still, in a critical review of conceptual and measurement inadequacies in this literature, Linda George wondered, perhaps with tongue in cheek, whether for this area of research it was “time to be born again” [57].

The challenge here in assessing religious attendance is not simply related to the metric used, but is more about the absence of a reasonable underlying theoretical justification [67,68]. Religious attendance has been called something of a “black box,” characterized by a complexity of “physical, sensory, behavioral, emotional, and cognitive experiences to which people are exposed when attending religious worship services” [69], p. 10, an exacerbating factor beyond the purely methodological that serves to complicate the construction of items or indices.

Regarding use of single-item measures of religious service attendance, consider these complexities just in reference to the Western monotheisms. Regular or expected church attendance for mainline Protestants may be once weekly, for Pentecostal or charismatic Christians may also include a weekly prayer service (e.g., on Wednesday) in addition to Sunday morning worship, and for Roman Catholics being a daily communicant may be encouraged as an ideal marker of piety. Among traditional Orthodox Jews attending synagogue prayer services thrice daily is expected, including for *shacharit* (morning), *minchah* (afternoon), and *maariv* (evening) prayer. Among Muslims, *salah* (ritual prayer) is obligated five times daily on a set time schedule, preferably in a *masjid* (mosque) but elsewhere if one is not accessible. In light of these norms, it may not make sense to use a more-than-weekly vs. less-than-weekly binary variable as a meaningful marker of religious participation much less of an overarching religiousness or faith or spirituality and, moreover, as a putative determinant of mental or even physical health. Because this is such a challenging conceptual issue, a research literature attempting to draw meaningful conclusions about risk and protection for morbidity or mortality due to religious involvement based primarily on whether one does such a thing more or less than once a week—or prays or reads scriptures or exhibits some other religious behavior or belief on a yes/no metric—would do well to take care not to overstate the universality of observed findings and to be more explicitly mindful of the limitations present in the content and metrics of particular measures. What is normative for some people or religious groups may be considered non-observance or even apostasy by another.

This situation presents a significant challenge for devising measures which can be applicable across religiously diverse populations. No easy solution is offered here, but greater sensitivity to this issue would benefit investigators and perhaps militate against the over-interpretation of findings. Findings observed among respondents in a particular population, representing a particular faith tradition, and according to a particular religious indicator do not enable us to draw broad conclusions about the instrumental function or value of religion, overall, for all people. A useful suggestion for epidemiologists might be to peruse the literature of validated religious measures, including multidimensional scales, as developed by sociologists and psychologists of religion, experts in religious measurement. A seminal collection, though by now quite dated, is found in Hill and Hood's classic *Measures of Religiosity* [70]. It may serve as a helpful baseline for exploring the subsequent literature on this topic and as a constructive source for creative ideas.

### Spirituality

Since the rise of public awareness of this research in the 1990s, a subset of clinical and population-health studies has appeared using the term spirituality as a substitute for or adjunct to religion or religiousness or religiosity. Spirituality is emerging as the preferred terminology within academic medicine [71], but this has become a source of some debate within medical research circles [72]. In traditional usage within academic religious studies and within the world's faith traditions when denoting a personal characteristic, the word spirituality has referenced what might be described as a transcendent or unitive or noetic sense or state of being [73] which is the result of a lifetime of religious observance or piety. As a result of hewing to the standards or strictures of particular religions, such as through one's behavior or beliefs, one might hope eventually, in this life or in the great beyond, to have attained a state of spirituality. It could be thought of as akin to an endpoint or objective or ultimate aim of a lifetime of faith, and is defined distinctively in respective religions. Alternatively, the word spirituality is typically used to label a respective pathway to such a state of being through following a particular religious or wisdom tradition [74]. That is, one may speak of Tibetan Buddhist spirituality, Anglican spirituality, Jewish spirituality, Taoist spirituality, Sufi spirituality, New Age spirituality, and so on. In this usage, the word references not just the end point, if you will, but the pathway to get there. These two uses of the word spirituality more or less define how the term has long been understood within religious and theological circles. How best to translate this into contemporary medical or epidemiologic research, however, including for use in religiously heterogeneous populations, has proven complex.

More recently, for example, within academic medicine and epidemiology, the words spiritual and spirituality have often been relied upon as a complement, substitute, or proxy for religious and religion. In this usage, spirituality is the larger, overarching term and religion denotes a smaller subset defining only involvement in institutional religions. Thus, one could claim to be “spiritual but not religious” [75], presumably with access to the health benefits of religion as observed in the volumes of epidemiologic studies noted earlier, but without a formal affiliation with an organized religious communion [76,77]. It remains an open question whether this conclusion has been validated, as most of the studies referenced here have been based on analyses of the morbidity or mortality outcomes of religious behaviors, beliefs, attitudes, experiences, and the like, rather than spirituality according to any definition, traditional or more contemporary. This presents a challenge—and an opportunity—for epidemiologists and other investigators who wish to probe the population-health impact of spirituality outside of an institutional religious context and to broach this topic with patient populations or survey respondents who may not formally identify with a religion [71].

While there have been hundreds of validated measures of dimensions and domains of religiousness, broadly defined, dating back to the 1960s and earlier [70], until recently there has been no consensus definition of this contemporary use of spirituality [78], nor any consensus conceptual development nor standard operationalization of the construct, as far as validated items, dimensions, and indices or scales. This is not to say that physicians, psychologists, and social scientists have not proffered definitions based on panels or other collaborative efforts [1,[79], [80], [81], [82], [83], [84]]. This work is commendable, although orthogonal to traditional usages by academic religious and theological scholars [85], as noted, but as yet has not influenced most usages of the term within medical or epidemiologic studies which persist in undefined uses which seem reflexive [72].

Most existing research studies in epidemiology, as summarized above, have been based on associations with religious identity or expression, not spirituality by any definition. This is despite the efforts in recent years to develop measures of the construct and to use them in studies, such as of psychiatric and chronic disease morbidity and general well-being [86]. Yet, on the whole, an often observed use of the term spirituality in medical and population-health resarch is simply as a proxy for the word religion, or as a hybrid form such as religion/spirituality or “R/S.” An additional complicating issue is the possibility of what has been termed “contamination” of spirituality measures, whereby indicators of mental health or well-being are incorporated into indices of the exposure variable, thus raising the possibility of positive findings which may be tautological [87,88].

These issues underscore what has been referred to as the “conceptual vagueness” [89] of much of the epidemiologic literature on religion. To be clear, the present author believes that more programmatic epidemiologic research on spirituality as an exposure construct, however defined, would be very welcomed, but, to repeat, this has not happened yet to any large extent, despite a tacit presumption of some laypeople and clinicians that published studies in this literature are all about spirituality. Encouragingly, recent efforts to upgrade the conceptual status of spiritual assessment for research on population health, psychology, and other fields have culminated in an excellent volume on the subject [90].

## Meaning

Notwithstanding potential issues with assessment of religious exposures, including spirituality, the overall weight of findings from existing studies suggests a generally protective effect. That is, lower levels of religious identity or belief or practice, whether assessed reliably and validly or not, seem to be associated with an elevated risk of morbidity and mortality. This has been observed consistently for decades [[1], [2], [3]], including, as noted, in analyses adjusting for exogenous factors such as age, sex, marital status, social class, race and ethnicity, urbanicity, and more. But as with all such findings, especially regarding the sorts of subjectively determined states assessed within psychosocial epidemiology, and certainly religious exposures qualify here, it is imperative to parse out what these findings mean and do not mean. The caveats noted earlier—that epidemiologists present associations that are observed on average and across populations [91], as well as adjusting as best we can for confounding or extraneous factors—are oftentimes lost on laypeople, and even on some clinicians. The risk here is that findings on this particular subject are typically presented to the public as “religion is good (or bad) for your health” [92], or results of one study are treated as a referendum on the truth claims of a particular faith or as an evangelistic spur to believe in God or in some sectarian religious tenet [93]. This is a very unfortunate misuse of scientific research, rightly critiqued by skeptics of religion [94], and, regardless, epidemiology is not equipped to address such existential questions. Laying out the meaning and non-meaning of these findings, especially their limitations, is thus an imperative task if this field of research is to continue maturing.

### Misinterpretation of findings

Through misinterpretation or overstatement, some proponents have misread these findings and drawn conclusions that do not derive from the data. Among the most commonly observed misinterpretations attributed to these findings [95] are that (a) religious involvement by itself (e.g., simply attending services or having religious beliefs; we are not speaking here of actual prayers for healing) and by some mysterious process is capable of healing, curing, or reversing pathology; (b) devoutly religious people do not (or should not) get sick, which would seem an untenable and even impossible claim on the surface; (c) religion or spirituality is the most important factor in health, something that study results do not support; and (d) these studies provide evidence of a supernatural influence on health. Regarding the latter, granted, according to many people of faith, “divine healing” may be possible, but if one is looking for validation of this belief from observational research of natural phenomena, such as through epidemiologic studies of morbidity rates in particular populations, then one is likely looking in the wrong place [96].

Published findings by epidemiologists reporting on associations between religious exposures and morbidity or morality outcomes in general (healthy) populations cannot be used as evidence for the healing of disease, divinely or otherwise. Many efforts have been made to clarify the distinctions between randomized clinical trials of healing prayer (of which there have been many, and which remain controversial [97,98]) and epidemiologic studies of religious indicators as putative risk or protective factors in relation to rates of morbidity and mortality in general populations [99]. Although this simple distinction is straightforward and of course understood by epidemiologists, it is sometimes overlooked or not fully understood in published overviews of the larger subject of faith and medicine [100]. To the point, while randomized clinical trials have purported to identify a healing sequela of prayer [98], these studies may or may not indicate some sort of divine or supernatural action [101], but this cannot be conclusively proven in the ways that other biomedical or epidemiologic research can since the source of the action (e.g., God), presumably, “resides” in whole or part outside of the natural world. There is a substantial conceptual and theoretical literature on this topic that may be unfamiliar to epidemiologists, and provocative summaries, pro and con, are available for interested readers [97,102].

As far as the results of observational studies of the types done by epidemiologists and sociomedical scientists, observed association between religious indicators and health outcomes should not be used to infer evidence of supernatural effects, whether or not such an effect is “really” operating. For one, these are two distinct types of studies (experimental trials of sick people vs. population surveys of general populations), and, secondly, neither epidemiology nor any other natural science is equipped to vet phenomena that, if real, have been described as wholly or partly non-naturalistic. A personal disclosure: the present author is a religious believer sympathetic to the idea of a deity who responds to prayer, but, to reiterate, population studies which identify, for example, a protective effect of weekly religious attendance on some cause-specific morbidity rate cannot possibly provide evidence for the healing or cure of disease, whether supernaturally gifted from God or naturalistic in origin. Efforts to make this claim are a substantial misuse of epidemiologic research.

Making this type of misattribution has served to fuel skepticism among clinicians and scientists [103], but, to be fair, both sides have misstated the meaning of findings: the “pro” side, as just noted, by earnestly reading more into these results than they merit, and the “con” side by dismissing the mass of validly obtained findings in part because of genuine unease over the subject matter. These stances may reflect understandable presuppositions or concerns, but it is important not to let either overenthusiasm about or disdain for religion or particular religions color an ability to view such findings more dispassionately.

### Mediators of observed associations

In deciphering what these findings do (or seem to) imply, it has proven helpful to explore possible mediators of a presumptive association between religiousness and health or disease, in general terms. Many taxonomies have been proposed [93,95,[104], [105], [106], [107]], and some of these mediators (or “mechanisms,” as they have come to be known in this literature) have been empirically validated. It is important to recognize that different dimensions or expressions of religious involvement may impact on health status through different means, as well as differently in different people or populations. For example, (a) religious commitment may lower disease risk and enhance well-being through motivating or reinforcing healthy life styles or health-related behaviors [108]; (b) religious fellowship may serve to buffer stress and enhance coping through offering supportive social relationships, in terms of the quantity and quality of both tangible and emotional support [109,110]; (c) religious worship, prayer, and meditation may exhibit salutary or salutogenic (healing) psychoneuroimmunological or psychophysiological effects [111,112]; (d) religious beliefs or worldviews may influence health through a consonance with certain health-promoting beliefs or cognitions or personality styles [[113], [114], [115]]; (e) religious faith may engender something akin to a placebo effect through encouraging optimism, hope, and positive expectations [116]; and (f) religious or spiritual experiences may stimulate certain salutary neurophysiological responses and unitive states of transcendence that reframe illness or even produce improved health [117,118]. Whether or not all of these hypothetical mechanisms are correct in all instances, in all people and populations, the key point here is that they entail naturalistic processes and do not invoke any forces or phenomena incoherent with current understandings of health, physiology, or the human body. Even among those in the epidemiologic profession who may not personally have a religious worldview, one might still accept in principle that religion or spirituality or faith could function salutarily in this way, through naturalistic means, in some people.

Taking into account the various caveats noted above, a general conclusion can be drawn: at the population level, rates of morbidity and mortality due to certain physical and mental health outcomes seem to vary across religious affiliations and by levels of religiousness, and in some instances these associations withstand adjustment for other (sociodemographic) factors. While, as noted above, no conclusive attribution can made of any salutogenic effect nor an etiologic status for religion nor a putative supernatural influence based on the existing literature of research findings from observational studies, nonetheless the religious or spiritual domain would seem to merit a place at the table among those psychosocial constructs which when assessed in epidemiologic studies have been found to be associated with rates of morbidity or mortality. This would seem to be a supportable take-away, notwithstanding some epidemiologists who may have good reason to consider such findings confusing or troubling [43].

Accordingly, an important and overlooked issue is the pressing need to examine those in whom religion is a source of harm, such as in terms of psychological distress. While the weight of published findings appears to be in a protective or primary-preventive direction, as this association has disseminated into public awareness relatively little attention has been given to the identity and characteristics of those who do not fit this general pattern, nor to the “dark side” of religion [119]. Such one-sidedness is typical of epidemiologic research in general for psychosocial and behavioral determinants of morbidity and mortality, exemplified, for example, by the relatively sparse attention paid to the presumably protective “Type B" construct in comparison with the voluminous literature on cardiovascular risks attributed to the Type A behavior pattern [120]. In this instance, it is the presumably protective polarity (religiousness) which receives attention, rather than the putative risk factor (non-religiousness).

This phenomenon is an observed side effect of the enthusiasm sometimes associated with findings related to any new risk or protective factor, but, in this case especially, it would be worthwhile to pay closer attention to the counter-examples or outliers. These might include, for example, those people who have been wounded or abused by religion, whether by one's congregation, one's clergy, or by particular beliefs or doctrines that make them feel mostly condemned and worthless. Respective religions prescribe and proscribe beliefs, attitudes, and practices that cover the gamut [121], some of which may fuel harmful ideations and thus produce deleterious psychological or physical outcomes. These experiences ought not be brushed aside, but should be tabulated, documented, and catalogued at the population level. This would be a worthy research frontier for this field moving forward.

## Implications

If there is by now an emerging sense of what these findings seem to mean and not mean, another key issue altogether is whether they matter. Specifically, does the observation of findings suggestive of health risk or protection attributable to one's status according to indicators of religious identity or practice actually matter for population-health research, intervention, or policy? Do these findings produced through epidemiologic and other empirical population-based research (e.g., medical sociology [122], social demography [123], health psychology [124]) have identifiable implications for the health and well-being of populations? It is being suggested here that the answer is a confident yes. There do seem to be substantive implications, and they constitute what could be considered a roadmap for a translational epidemiology of religion [125].

First, there are implications for *epidemiologic and population-health research*. This includes studies of the determinants of morbidity and mortality and evaluative research on community health interventions. The empirical religion and health literature would seem to suggest that it may be necessary to incorporate religious indicators as main effects or covariates if we are to avoid misspecifying both predictive models of the determinants of health [126] and theoretical models guiding public health interventions [127]. Just as certain sociodemographic, behavioral, and medical-history variables are now tacitly included in such models, there is sufficient evidence that religiousness, variously assessed, is a determinant or class of determinants whose effects ought to be investigated or adjusted for in studies [128].

Second, there are implications for *clinical medicine*, in both institutional and community settings [129]. Religious factors may substantively impact on various parameters of the illness and medical care experience, such as the course of treatment, prognosis, length of hospital stays, patient satisfaction, and whether there are psychological or psychiatric sequelae of chronic or acute illness experiences, and on utilization rates in certain populations [130]. These observed associations ought to alert clinicians to the need for sensitive spiritual assessment and pastoral referrals [131] in order to identify and accommodate the possible religious needs of patients. For people of faith, illness and especially hospitalization may entail being separated from one's faith community, from one's source of meaning and sustenance, and, by itself, this can exacerbate pathology or delay recovery [132]. Medical care providers need to be aware of this possibility and of evidence from research studies which are supportive of compassionate patient-centered care [133].

Third, there are implications for planning *congregational health promotion and disease prevention programs*. Besides considering religious influences when evaluating interventions, loci of religious activity and fellowship—namely churches, synagogues, mosques, temples, and other places of worship—may be ideal sites for implementing health outreach programs for preventing disease and promoting well-being [134]. There is a tradition of such programming in the U.S. dating to the 1970s [135], and this work has proven especially valuable in reaching underserved communities and populations in whom religious institutions may be the primary autonomous organization of respective at-risk groups of people [136]. If empirical evidence suggests that religious participation is health-impacting, then programs of health outreach (e.g., free clinics, preventive services, screening) that connect with people through their congregations and which reinforce decisions to attend services may be invaluable means to address population-health disparities [137].

Fourth, there are implications for *global health development and public health policy*. Domestically and globally, national health ministries, the World Health Organization, and numerous nongovernmental organizations, especially in the developing world, have in place governmental, civil-society-sector, and philanthropic initiatives to draw on faith-based organizations to bolster the public health infrastructure of respective countries and to address acute challenges that may arise [138]. Such programs and initiatives are sources of health-impacting religious and social capital and their ongoing support is a function of the involvement of people committed to externalizing the moral teachings of respective religions [139]. Adherence to such teachings also serves as an underpinning of efforts to influence national public health policies, such as the widespread religious advocacy that impacted the direction of the healthcare reform debate in the U.S. in the early 2000s [140].

Fifth, there are implications for *human flourishing*. This construct, or rather meta-construct, has gained considerable currency in recent years as an outcome encompassing a variety of health-, well-being-, and quality-of-life-related dimensions, including physical and mental health; happiness and life satisfaction; meaning and purpose; close social relationships; and character and virtue [141]. Findings from population-based research studies implicate religiousness, in a broad sense, as a protective or promotive factor in each of these domains. This has drawn the attention of the academic, philanthropic, and governmental sectors, and, at present, a variety of initiatives are emerging which draw on this work to advance the concept of flourishing in various settings, from the workplace to healthcare institutions to the economic system to global governance [142]. Especially notable is the Global Flourishing Study, which has sought to create a series of religious items amenable to global comparative research [[143], [144], [145]] and which, among other published results, has produced population-based findings on religious and health indicators and the associations between them from a 22-nation sample [146].

## Conclusions

In sum, over a century of population-health research has accumulated providing empirical evidence of a connection between religious identity and participation and rates of morbidity and mortality. Limitations in assessment of religious exposures, in providing coherent explanations for putative religious associations with health outcomes, and in detailing the larger implications of these findings for public health have presented challenges for the widespread acceptance of this work among epidemiologists and clinicians. More recent studies, for the most part, have been methodologically sound and do a better job at identifying and adjusting for potential mediating constructs and pointing to research, intervention, and policy implications. Still, investigators must continue to up the bar on detailing what these findings mean and why they matter.

In 1988, Leon Gordis famously stated,

There should … be tolerance or indeed enthusiasm for unorthodox ideas. When a discipline is willing to support only an accepted way of thinking and an established dogma, its very health becomes threatened. We should never allow ourselves to reach a point where epidemiology no longer fosters innovation in the form of imaginative hypotheses [147].

This makes a nice proof text for investigating unusual exposure constructs and variables, and for epidemiology certainly religion qualifies. But it places the burden on those conducting this research to ensure that their religious measures are validated and are coherent with the norms of the populations being studied, and that any findings which emerge are carefully vetted, with potential mediators documented or proposed and with real-world implications for population health laid out clearly.

## CRediT authorship contribution statement

**Jeff Levin:** Writing – review & editing, Writing – original draft, Conceptualization.

## Ethics approval

Ethics approval was not needed because this is a commentary article and no human patients were involved.

## Funding statement

No funding was received in support of the author's work on this paper.

## Declaration of competing interest

The author declares that there are no conflicts of interest.

## Data Availability

No new data were generated or analyzed in support of this review article.

## References

[1] Koenig H.G., VanderWeele T.J., Peteet J.R. (2024).

[2] Levin J.S., Schiller P.L. (2025). Is there a religious factor in health?. J Relig Health.

[3] Jarvis G.K., Northcott H.C. (1987). Religion and differences in morbidity and mortality. Soc Sci Med.

[4] Zielhuis G.A., Kiemeney L.A. (2001). Social epidemiology? No way. Int J Epidemiol.

[5] Sloan R.P., Bagiella E., Powell T. (1999). Religion, spirituality, and medicine. Lancet.

[6] Flannelly K.J., Ellison C.G., Strock A.L. (2004). Methodologic issues in research on religion and health. South Med J.

[7] VanderWeele T.J. (2017). Religion and health in Europe: cultures, countries, context. Eur J Epidemiol.

[8] VanderWeele T.J., Balboni T.A., Koh H.K. (2017). Health and spirituality. JAMA.

[9] Larson D.B., Pattison E.M., Blazer D.G., Omran A.R., Kaplan B.H. (1986). Systematic analysis of research on religious variables in four major psychiatric journals, 1978-1982. Am J Psychiatry.

[10] Witter R.A., Stock W.A., Okun M.A., Haring M.J. (1985). Religion and subjective well-being in adulthood: a quantitative synthesis. Rev Relig Res.

[11] Cheng C., Ying W. (2023). A meta-analytic review of the associations between dimensions of religious coping and psychological symptoms during the first wave of the COVID-19 pandemic. Front Psych.

[12] Levin J.S., Vanderpool H.Y. (1987). Is frequent religious attendance *really* conducive to better health?: toward an epidemiology of religion. Soc Sci Med.

[13] Billings J.S. (1891). Vital statistics of the Jews. North Am Rev.

[14] Kennaway E.L. (1948). The racial and social incidence of cancer of the uterus. Br J Cancer.

[15] Seidman H. (1966). Lung cancer among Jewish, Catholic and Protestant males in New York City. Cancer.

[16] Srole L., Langner T. (1962). Mental health in the metropolis: The Midtown Manhattan Study.

[17] Blazer D., Palmore E. (1976). Religion and aging in a longitudinal panel. Gerontol.

[18] Rouse I.L., Armstrong B.K., Beilin L.J. (1982). Vegetarian diet, lifestyle and blood pressure in two religious populations. Clin Exp Pharmacol Physiol.

[19] Gillum R.F., King D.E., Obisesan TO, Koenig H.G. (2008). Frequency of attendance at religious services and mortality in a U.S. national cohort. Ann Epidemiol.

[20] King H., Bailar J.C. (1969). The health of the clergy: a review of demographic literature. Demogr.

[21] Levin J., Schaie K.W., Krause N., Booth A. (2004). Religious influences on health and well-being in the elderly.

[22] Silton N.R., Flannelly K.J., Galek K., Ellison C.G. (2014). Beliefs about God and mental health among American adults. J Relig Health.

[23] Stroope S., Kent B.V., Zhang Y., Kandula N.R., Kanaya A.M., Shields A.E. (2020). Self-rated religiosity/spirituality and four health outcomes among U.S. south Asians: findings from the Study on Stress, Spirituality, and Health. J Nerv Ment Dis.

[24] Koenig H.G., George L.K., Meador K.G., Blazer D.G., Ford S.M. (1994). Religious practices and alcoholism in a southern adult population. Psychiatr Serv.

[25] Johnson M.W., Hendricks P.S., Barrett F.S., Griffiths R.R. (2019). Classic psychedelics: an integrative review of epidemiology, therapeutics, mystical experience, and brain network function. Pharmacol Ther.

[26] Comstock G.W., Partridge K.B. (1972). Church attendance and health. J Chronic Dis.

[27] Koenig H.G., McCullough M.E., Larson D.B. (2001).

[28] Koenig H.G., King D.E., Carson V.B. (2012).

[29] Levin J., Levin J. (2018). Religion and the social sciences: Basic and applied research perspectives.

[30] Nakagawa S., Lagisz M., Yang Y., Drobniak S.M. (2024). Finding the right power balance: better study design and collaboration can reduce dependence on statistical power. PLoS Biol.

[31] Nirwan M., Halder K., Saha M., Pathak A., Balakrishnan R., Ganju L. (2021). Improvement in resilience and stress-related blood markers following ten months yoga practice in Antarctica. J Altern Complement Med.

[32] Hamman R.F., Barancik J.I., Lilienfeld A.M. (1981). Patterns of mortality in the old order Amish: I. Background and major causes of death. Am J Epidemiol.

[33] Dalgalarrondo P., Marín-León L., Botega N.J., De Azevedo Barros M.B., Bosco de Oliveira H. (2008). Religious affiliation and psychiatric morbidity in Brazil: higher rates among Evangelicals and Spiritists. Int J Soc Psychiatry.

[34] Kohler I.V., Preston S.H. (2011). Ethnic and religious differentials in Bulgarian mortality, 1993-98. Popul Stud.

[35] Jussawalla D.J., Jain D.K. (1977). Breast cancer and religion in greater Bombay women: an epidemiological study of 2130 women over a 9-year period. Br J Cancer.

[36] Scotch N.A. (1963). Sociocultural factors in the epidemiology of Zulu hypertension. Am J Public Health.

[37] Krause N., Ingersoll-Dayton B., Liang J., Sugisawa H. (1999). Religion, social support, and health among the Japanese elderly. J Health Soc Behav.

[38] Levin J.S. (1994). Religion and health: is there an association, is it valid, and is it causal?. Soc Sci Med.

[39] Troyer H. (1988). Review of cancer among 4 religious sects: evidence that life-styles are distinctive sets of risk factors. Soc Sci Med.

[40] Kaplan B.H. (1976). A note on religious beliefs and coronary heart disease. J S Car Med Ass.

[41] Levin J.S., Vanderpool H.Y. (1989). Is religion therapeutically significant for hypertension?. Soc Sci Med.

[42] Mindel C.H., Vaughan C.E. (1978). A multidimensional approach to religiosity and disengagement. J Gerontol.

[43] Sherrill K.A., Larson D.B., Levin J.S. (1994). Religion in aging and health: Theoretical foundations and methodological frontiers.

[44] Rāsānen J., Kauhanen J., Lakka T.A., Kaplan G.A., Salonen J.T. (1996). Religious affiliation and all-cause mortality: a prospective population study in middle-aged men in eastern Finland. Int J Epidemiol.

[45] Gartner J., Larson D.B., Allen G.D. (1991). Religious commitment and mental health: a review of the empirical literature. J Psychol Theol.

[46] Strawbridge W.J., Cohen R.D., Shema S.J., Kaplan G.A. (1997). Frequent attendance at religious services and mortality over 28 years. Am J Public Health.

[47] Graham T.W., Kaplan B.H., Cornoni-Huntley J.C., James S.A., Becker C., Hames C.G. (1978). Frequency of church attendance and blood pressure elevation. J Behav Med.

[48] House J.S., Robbins C., Metzner H.L. (1982). The association of social relationships and activities with mortality: prospective evidence from the Tecumseh Community Health Study. Am J Epidemiol.

[49] Williams D.R., Larson D.B., Buckler R.E., Heckmann R.C., Pyle C.M. (1991). Religion and psychological distress in a community sample. Soc Sci Med.

[50] Chen Y., Kim E.S., VanderWeele T.J. (2020). Religious-service attendance and subsequent health and well-being throughout adulthood: evidence from three prospective cohorts. Int J Epidemiol.

[51] Miller L., Wickramaratne P., Gameroff M.J., Sage M., Tenke C.E., Weissman M.M. (2012). Religiosity and major depression in adults at high risk: a ten-year prospective study. Am J Psychiatry.

[52] Idler E.L., Kasl S.V. (1997). Religion among disabled and nondisabled persons II: attendance at religious services as a predictor of the course of disability. J Gerontol B Psychol Sci Soc Sci.

[53] Park C.L., George J.R., Awao S., Carney L.M., Batt S., Salsman J.M. (2022). U.S. federal investment in religiousness/spirituality and health research: a systematic review. Religions.

[54] Markides K.S., Levin J.S., Ray L.A. (1987). Religion, aging, and life satisfaction: an eight-year, three-wave longitudinal study. Gerontol.

[55] Taylor R.J., Chatters L.M., Levin J. (2004).

[56] Armstrong B.K., White E., Saracci R. (1992).

[57] George L.K., Shah T.S., Friedman J. (2018). Homo religiosus?: Exploring the roots of religion and religious freedom in human experience.

[58] Krause N. (1997). Religion, aging, and health: current status and future prospects. J Gerontol B Psychol Sci Soc Sci.

[59] Gorsuch R.L. (1984). Measurement: the boon and bane of investigating religion. Am Psychol.

[60] Hall D.E., Koenig H.G., Meador K.G. (2004). Conceptualizing “religion”: how language shapes and constrains knowledge in the study of religion and health. Perspect Biol Med.

[61] Hall D.E., Meador K.G., Koenig H.G. (2008). Measuring religiousness in health research: review and critique. J Relig Health.

[62] Shuman J.J., Meador K.G. (2002).

[63] Williams D.R., Levin J.S. (1994). Religion in aging and health: Theoretical foundations and methodological frontiers.

[64] McCullough M.E., Larson D.B., Koenig H.G., Lerner R. (1999). The mismeasurement of religion in mortality research. Mortality.

[65] Zuckerman D.M., Kasl S.V., Ostfeld A.M. (1984). Psychosocial predictors of mortality among the elderly poor: the role of religion, well-being, and social contacts. Am J Epidemiol.

[66] Ransome Y. (2020). Religion, spirituality, and health: new considerations for epidemiology. Am J Epidemiol.

[67] Levin J., Chatters L.M., Taylor R.J. (2011). Theory in religion, aging, and health: an overview. J Relig Health.

[68] Krause N.M. (2022).

[69] Idler E.L., Boulifard D.A., Labouvie E., Chen Y.Y., Krause T.J., Contrada R.J. (2009). Looking inside the black box of “attendance at services”: new measures for exploring an old dimension in religion and health research. Int J Psychol Religion.

[70] Hill P.C., Hood R.W. (1999). Measures of religiosity.

[71] Balboni T.A., VanderWeele T.J., Doan-Soares S.D., Long K.N., Ferrell B.R., Fitchett G. (2022). Spirituality in serious illness and health. JAMA.

[72] Levin J. (2009). “And let us make us a name”: reflections on the future of the religion and health field. J Relig Health.

[73] Goring R. (1992). The Wordsworth dictionary of beliefs and religions.

[74] Van Ness P.H. (1996). Spirituality and the secular quest.

[75] Zinnbauer B.J., Pargament K.I., Cole B., Rye M.S., Butter E.M., Belavich T.G. (1997). Religion and spirituality: unfuzzying the fuzzy. J Sci Study Relig.

[76] Marty M.E. (2005). The religious foundations of law. Emory Law J.

[77] Smith H. (2001).

[78] Rose S. (2001). Is the term “spirituality” a word that everyone uses, but nobody knows what anyone means by it?. J Contemp Relig.

[79] Long K.N., Symons X., VanderWeele T.J., Balboni T.A., Rosmarin D.H., Puchalski C. (2024). Spirituality as a determinant of health: emerging policies, practices, and systems: article examines spirituality as a social determinant of health. Health Aff.

[80] Puchalski C.M., Blatt B., Kogan M., Butler A. (2014). Spirituality and health: the development of a field. Acad Med.

[81] Puchalski C.M., Vitillo R., Hull S.K., Reller N. (2014). Improving the spiritual dimension of whole person care: reaching national and international consensus. J Palliat Med.

[82] Pargament K.I., Pargament K.I., Exline J.J., Jones J.W. (2013). APA handbook of psychology, religion, and spirituality.

[83] Steinhauser K.E., Fitchett G., Handzo G.F., Johnson K.S., Koenig H.G., Pargament K.I. (2017). State of the science of spirituality and palliative care research part I: definitions, measurement, and outcomes. J Pain Symptom Manage.

[84] Ammerman N.T. (2013). Spiritual but not religious?: beyond binary choices in the study of religion. J Sci Study Relig.

[85] Marty M., Foster R.J. (1998). Streams of living water: Celebrating the great traditions of Christian faith.

[86] Underwood L.G., Teresi J.A. (2002). The Daily Spiritual Experience Scale: development, theoretical description, reliability, exploratory factor analysis, and preliminary construct validity using health-related data. Ann Behav Med.

[87] Koenig H.G., Carey L.B. (2024). Religion, spirituality and health research: warning of contaminated scales. J Relig Health.

[88] Koenig H.G. (2008). Concerns about measuring “spirituality” in research. J Nerv Ment Dis.

[89] Van Ness P.H. (2003). Epidemiology and the study of religion. Religion.

[90] Ai A.L., Wink P., Paloutzian R.F., Harris K.A. (2012). Assessing spirituality in a diverse world.

[91] D’Onise K. (March 7, 2025). https://katinadonise.com.au/post/epidemiology-moving-from-anecdotes-to-averages/.

[92] Roberts N.F. (2019). https://www.forbes.com/sites/nicolefisher/2019/03/29/science-says-religion-is-good-for-your-health/.

[93] Ellison C.G., Levin J.S. (1998). The religion-health connection: evidence, theory, and future directions. Health Educ Behav.

[94] Sloan R.P. (2006).

[95] Levin J.S. (1996). How religion influences morbidity and health: reflections on natural history, salutogenesis and host resistance. Soc Sci Med.

[96] Levin J. (2010). Religion and mental health: theory and research. Int J Appl Psychoanal Stud.

[97] Dossey L., Hufford D.J. (2005). Are prayer experiments legitimate?: twenty criticisms. Explore (NY).

[98] VanderWeele T.J., Peteet J.R., Balboni J.M. (2017). Spirituality and medicine within the culture of medicine: From evidence to practice.

[99] Levin J., Levin J. (2020). Religion and medicine: A history of the encounter between humanity’s two greatest institutions.

[100] Levin J. (2018). The discourse on faith and medicine: a tale of two literatures. Theor Med Bioeth.

[101] Levin J.S. (1996). How prayer heals: a theoretical model. Alt Ther Health Med.

[102] VandeCreek L. (1998). Scientific and pastoral perspectives on intercessory prayer: An exchange between Larry Dossey, M.D. and health care chaplains.

[103] Koenig H.G., Idler E., Kasl S., Hays J.C., George L.K., Musick M. (1999). Religion, spirituality, and medicine: a rebuttal to skeptics. Int J Psychiatry Med.

[104] Idler E.L. (1987). Religious involvement and the health of the elderly: some hypotheses and an initial test. Soc Forces.

[105] McIntosh D., Spilka B. (1990). Religion and physical health: the role of personal faith and control beliefs. Res Soc Sci Study Relig.

[106] George L.K., Ellison C.G., Larson D.B. (2002). Explaining the relationships between religious involvement and health. Psychol Inq.

[107] Ellison C.G., Levin J.S. (1994). Religion in aging and health: Theoretical foundations and methodological frontiers.

[108] Hill T.D., Ellison C.G., Burdette A.M., Musick M.A. (2007). Religious involvement and healthy lifestyles: evidence from the Survey of Texas Adults. Ann Behav Med.

[109] Krause N. (2008).

[110] Strawbridge W.J., Shema S.J., Cohen R.D., Kaplan G.A. (2001). Religious attendance increases survival by improving and maintaining good health behaviors, mental health, and social relationships. Ann Behav Med.

[111] Murphy M., Donovan S., Taylor E. (1997).

[112] Koenig H.G., Cohen H.J. (2002). The link between religion and health: Psychoneuroimmunology and the faith factor.

[113] Dull V.T., Skokan L.A. (1995). A cognitive model of religion’s influence on health. Aust J Soc Issues.

[114] James A., Wells A. (2003). Religion and mental health: towards a cognitive-behavioural framework. Br J Health Psychol.

[115] McCullough M.E., Willoughby B.L. (2009). Religion, self-regulation, and self-control: associations, explanations, and implications. Psychol Bull.

[116] Levin J. (2008). How faith heals: A theoretical model. Explore (NY).

[117] Levin J., Steele L., Levin J., Steele L. (2005). The transcendent experience: conceptual, theoretical, and epidemiologic perspectives. Explore (NY).

[118] Levin J. (2023). Nothingness, oneness, and infinity: transcendent experience as a promising frontier for religion and health research. J Relig Health.

[119] Krause N., Wulff K.M. (2004). Religious doubt and health: exploring the potential dark side of religion. Sociol Relig.

[120] Kaplan B.H. (1992). Social health and the forgiving heart: the Type B story. J Behav Med.

[121] Vaux K. (1976). Religion and health. Prev Med.

[122] Ellison C.G. (1995). Race, religious involvement and depressive symptomatology in a southeastern U.S. community. Soc Sci Med.

[123] Hummer R.A., Rogers R.G., Nam C.B., Ellison C.G. (1999). Religious involvement and U.S. adult mortality. Demogr.

[124] Park C.L. (2007). Religiousness/spirituality and health: a meaning systems perspective. J Behav Med.

[125] Levin J. (2022). Toward a translational epidemiology of religion: challenges and applications. Ann Epidemiol.

[126] Kawachi I. (2020). Invited commentary: religion as a social determinant of health. Am J Epidemiol.

[127] Idler E.L. (2014). Religion as a social determinant of public health.

[128] Idler E., Cochrane J.R., Gunderson G., Cutts T. (2024). Handbook on religion and health: Pathways for a turbulent future.

[129] VanderWeele T.J., Balboni T.A., Koh H.K. (2022). Invited commentary: religious service attendance and implications for clinical care, community participation, and public health. Am J Epidemiol.

[130] Schiller P.L., Levin J.S. (1988). Is there a religious factor in health care utilization? A review. Soc Sci Med.

[131] Koenig H.G. (2001). Religion and medicine IV: religion, physical health, and clinical implications. Int J Psychiatry Med.

[132] Koenig H.G. (2014). The spiritual care team: enabling the practice of whole person medicine. Religions.

[133] Post S.G. (2011). Compassionate care enhancement: benefits and outcomes. Int J Pers Cent Med.

[134] Kegler M.C., Hall S.M., Kiser M. (2010). Facilitators, challenges, and collaborative activities in faith and health partnerships to address health disparities. Health Educ Behav.

[135] Idler E., Levin J., VanderWeele T.J., Khan A. (2019). Partnerships between public health agencies and faith communities. Am J Public Health.

[136] Eng E., Hatch J., Callan A. (1985). Institutionalizing social support through the church and into the community. Health Ed Q.

[137] Campbell M.K., Hudson M.A., Resnicow K., Blakeney N., Paxton A., Baskin M. (2007). Church-based health promotion interventions: evidence and lessons learned. Annu Rev Public Health.

[138] Winiger F., Peng-Keller S. (2021). Religion and the World Health Organization: an evolving relationship. BMJ Glob Health.

[139] Gunderson G.R., Cochrane J.R. (2012).

[140] Cammisa A.M., Manuel P.C. (2016). Religious groups as interest groups: the United States Catholic bishops in the welfare reform debate of 1995-1996 and the health care reform debate of 2009-2010. Religions.

[141] VanderWeele T.J. (2017). On the promotion of human flourishing. Proc Natl Acad Sci U S A.

[142] Lomas T., Pawelski J.O., VanderWeele T.J. (2023). A flexible map of flourishing: the dynamics and drivers of flourishing, well-being, health, and happiness. Int J Wellbeing.

[143] Johnson K.A., Moon J.W., VanderWeele T.J., Schnitker S., Johnson B.R. (2024). Assessing religion and spirituality in a cross-cultural sample: development of religion and spirituality items for the Global Flourishing Study. Relig Brain Behav.

[144] Lomas T., Bradshaw M., Case B., Cowden R.G., Crabtree S., English C. (2020). The development of the Global Flourishing Study questionnaire: charting the evolution of a new 109-item inventory of human flourishing. BMC Glob Public Health.

[145] Crabtree S., English C., Johnson B.R., Ritter Z., VanderWeele T.J. (2025). https://osf.io/y3t6m.

[146] Levin J., Bradshaw M., Johnson B.R. (2025). Association between Jewish religious observance and mental health among Israeli adults: findings from the Global Flourishing Study. Int J Psychiatry Med.

[147] Gordis L. (1988). Challenges to epidemiology in the next decade. Am J Epidemiol.

